# Converging on a core cognitive deficit: the impact of various neurodevelopmental insults on cognitive control

**DOI:** 10.3389/fnins.2014.00153

**Published:** 2014-06-11

**Authors:** Kally C. O'Reilly, Hsin-Yi Kao, Heekyung Lee, André A. Fenton

**Affiliations:** ^1^Neurobiology of Cognition Laboratory, Center for Neural Science, New York UniversityNew York, NY, USA; ^2^Graduate Program in Neural and Behavioral Science, Downstate Medical Center, State University of New YorkBrooklyn, NY, USA; ^3^The Robert F. Furchgott Center in Neural and Behavioral Science, Downstate Medical Center, State University of New YorkBrooklyn, NY, USA

**Keywords:** cognitive control, neurodevelopmental insults, mental illness, hippocampus, neural coordination, schizophrenia models

## Abstract

Despite substantial effort and immense need, the treatment options for major neuropsychiatric illnesses like schizophrenia are limited and largely ineffective at improving the most debilitating cognitive symptoms that are central to mental illness. These symptoms include cognitive control deficits, the inability to selectively use information that is currently relevant and ignore what is currently irrelevant. Contemporary attempts to accelerate progress are in part founded on an effort to reconceptualize neuropsychiatric illness as a disorder of neural development. This neuro-developmental framework emphasizes abnormal neural circuits on the one hand, and on the other, it suggests there are therapeutic opportunities to exploit the developmental processes of excitatory neuron pruning, inhibitory neuron proliferation, elaboration of myelination, and other circuit refinements that extend through adolescence and into early adulthood. We have crafted a preclinical research program aimed at cognition failures that may be relevant to mental illness. By working with a variety of neurodevelopmental rodent models, we strive to identify a common pathophysiology that underlies cognitive control failure as well as a common strategy for improving cognition in the face of neural circuit abnormalities. Here we review our work to characterize cognitive control deficits in rats with a neonatal ventral hippocampus lesion and rats that were exposed to Methylazoxymethanol acetate (MAM) *in utero*. We review our findings as they pertain to early developmental processes, including neurogenesis, as well as the power of cognitive experience to refine neural circuit function within the mature and maturing brain's cognitive circuitry.

## Introduction

Schizophrenia is a complex mental disorder with varying degrees of severity. One of the most debilitating deficits in schizophrenia patients is impoverished cognitive control, the inability to ignore irrelevant information while using relevant information to complete a task (Nuechterlein et al., 2008). We have chosen to utilize three neurodevelopmental rodent models to examine the hypothesis that poor cognitive control is a result of failed neural coordination, manifest as inappropriate temporal organization of neural electrical activity within and between neural circuits. This neural discoordination hypothesis is agnostic to the etiology of the discoordination. Furthermore, the hypothesis predicts that if neural coordination can be restored, cognitive function will also be restored. We are investigating this hypothesis using different neurodevelopmental models because the disease is increasingly thought to have diverse neurodevelopmental origins. Furthermore, because altering neurodevelopment likely alters a host of nonspecific, or even unidentified factors within the brain, the ability to overcome the functional consequences of abnormal development is quite compelling, especially if compensation for neural discoordination may be achieved without having to target, study, or reverse the original neurodevelopmental insult.

## Cognitive control

### Cognitive control and neural coordination

**Figure 1:**
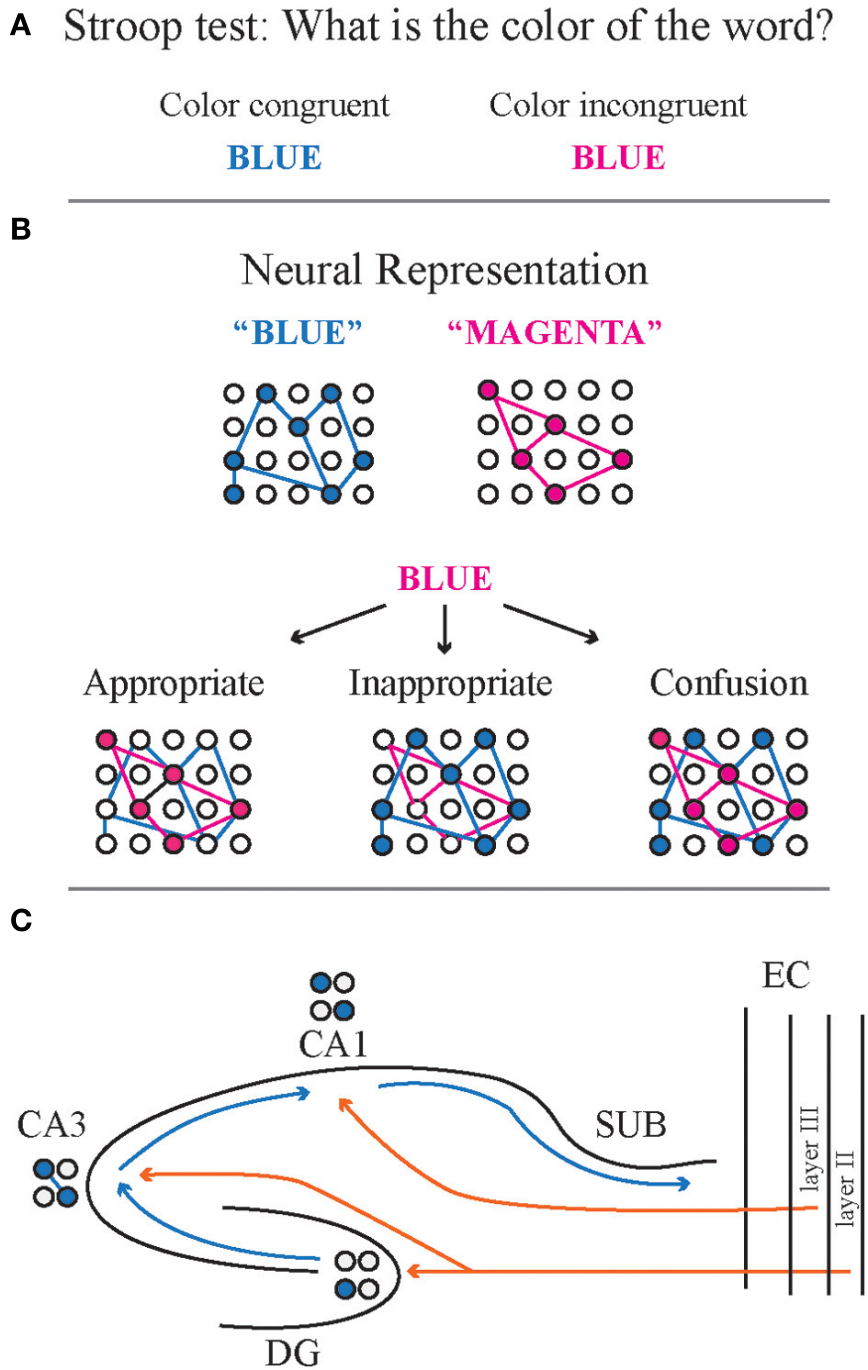
**Cognitive control depends on neural coordination, the coordinated activity of neural networks within or among brain regions. (A)** During the Stroop test, the test subject is presented with a word that means a color. The word meaning and color match for color-meaning congruent words, whereas there is a mismatch for color incongruent words. **(B)** Neural representations are a coactive ensemble of neurons that coalesce into a temporally defined pattern to represent a concept. Neural coordination to express the appropriate representation is required for appropriate responds during the Stroop test. Coactivity implies that the neurons are functionally coupled to drive the emergence of the ensemble. The cognitive challenge is escalated for presentations of color incongruent words because the appropriate response requires suppression of the inappropriate neural representation, despite the presence of functional coupling to drive the neural representation for the word name. The appropriate response contrasts with the case of an inappropriate response to the color-incongruent word. Instead of the neural representation of the color, the inappropriate representation of the meaning emerges, suppressing the appropriate representation of the color. The neural equivalent of confusion results when both the color and meaning representations are simultaneously active, which is altogether a different inappropriate representation. **(C)** The hippocampus is a complex circuit in which multiple streams of information converge on a single area. For example, information from CA3 and entorhinal cortex must be coordinated in order to activate the appropriate response in CA1, a point of convergence for CA3 and EC activity. The dominant contemporary model asserts that the dentate gyrus mediates pattern separation because a few, sparsely organized neurons are activated to relay incoming cortical information to CA3. CA3 mediates pattern completion because the recurrent collaterals provides for relatively high autoassociative connectivity, and a less sparse pattern of activation. This means that the CA3 output will minimize differences between different representations once they are small and the differences will be exaggerated if they are large. The CA1 subfield is thought to relay the result of the “processed” combined dentate gyrus and CA3 computations to the neocortex via the subiculum. Given that CA1 also receives direct neocortical input, this subfield may function to integrate and compare the two direct and processed inputs. CA1, cornu ammonis 1 subregion; CA3, cornu ammonis 3 subregion; DG, dentate gyrus; SUB, subiculum; EC, entorhinal cortex.

Our lab focuses on behaviors that require cognitive control, the process by which relevant information is used, and irrelevant information ignored in the service of a subject's goals. The Stroop test is a canonical test for cognitive control (Stroop, 1935). Subjects can be presented a written word whose meaning is a color. The subject is instructed to name the color in which the word is written and ignore the meaning of the word. In its basic form, the word for a color (e.g., blue) is presented in either the same color (blue) or a different color (e.g., magenta) (Figure 1A). Congruent word-color presentations require minimal cognitive control, whereas the color incongruent presentations require cognitive control to ignore the meaning of the word, which is the prepotent response, and instead report the color in which the word was written. Patients with schizophrenia are impaired at responding to color incongruent presentations and they respond faster to color congruent words than controls (Carter et al., 1992; Perlstein et al., 1998). In a sense, all purposeful experience requires the ability to select for processing the information that is relevant and filter out or ignore what is currently irrelevant, similar to how all such abilities rely on memory. The concept of cognitive control, like memory, as it is studied, however, is more specific. Cognitive control is primarily concerned with the effortful top-down cognitive process (Miller and Cohen, 2001) that biases representations and actions in accord with internal goals. Since cognitive control is not itself an overt action, and like memory, it can only be currently inferred from behavior, it is important to have a clear operational definition of cognitive control along with evaluative tests that can systematically vary the demand for this ability. As we learn more about the neurobiological basis of these psychological concepts it may be possible to define the concepts in terms of their underlying biology (Kelemen and Fenton, 2010).

Our work with rodents is centered on cognitive control because poor cognitive control is a fundamental deficit in schizophrenia and other mental illness, and a good predictor of functional outcome (Carter and Barch, 2007). In addition, cognitive control creates experimental conditions that can help elucidate the form of the neural code that a circuit may be using. Indeed, how neurons code information is a fundamental and open question. The two major hypotheses are dedicated and ensemble coding. Dedicated coding, analogous to the green light in a traffic sign, is based on the concept of cardinal cells (e.g., Barlow, 1972). These are neural units dedicated to signaling high-order stimuli and concepts, like “face” or “grandmother.” Single cells that respond to individuals like “Jennifer Aniston” and specific video clips have been recorded from people (Quiroga et al., 2005; Gelbard-Sagiv et al., 2008), although this may not be sufficiently compelling evidence of a dedicated code (Quiroga et al., 2008).

Our work is founded in the alternative, ensemble coding hypothesis. Ensemble coding uses the activity of a group of cells to encode information (Figure 1B). It is analogous to a stadium jumbotron that uses many lights to encode a message. More messages can be encoded than there are lights, and no single light is essential for a message. Hebb's (1949) cell assembly postulate is an ensemble coding scheme. A cell assembly is a set of linked cells that fire together to represent information. Ensemble coding must avoid the loss of information that would result from conjointly activating multiple representations with overlapping cells, which has been called the superposition catastrophe (Von Der Malsburg and Schneider, 1986). Just as a jumbotron cannot simultaneously display two messages with the same lights, cell assemblies cannot coactivate without mechanisms to appropriately subgroup their conjoint activity. Without effective grouping mechanisms, the coactive assemblies merge into one assembly with catastrophic information loss (“Confusion” in Figure 1B).

One way to avoid the superposition catastrophe when two distinct items need to be represented in a structure is to alternate between activating one representation, then the other (Figure 1B). This mechanism, called temporal or dynamic grouping, may occur on the gamma (40 ms) timescale in neocortex (see Singer, 1999), and the gamma and theta (~140 ms) timescales in hippocampus (Buzsaki, 2010). In fact, during the active place avoidance tasks that we employ, dynamic grouping has been observed in the discharge of rat hippocampal place cells (Kelemen and Fenton, 2010) at multiple time scales from 25 ms to several seconds (Kelemen and Fenton, 2012, 2013).

Straightforward evidence of the hippocampus using an ensemble place code comes from the study of hippocampal place cells. A place cell discharges selectively when a rat is in a portion of the environment called the cell's firing field (O'Keefe and Dostrovsky, 1971; Muller et al., 1987). Place cell firing fields tend to be stable across days (Muller et al., 1987; Thompson and Best, 1990) although they only intermittently participate in the active subset of the hippocampus representation of an environment on the timescales of seconds (Fenton and Muller, 1998; Fenton et al., 2010) and days (Ziv et al., 2013), phenomena consistent with ensemble coding. In addition to this temporal uncertainty, there is also fundamental spatial uncertainty, because place cells in dentate gyrus, CA3 and CA1 have multiple firing fields in sufficiently large environments. This indicates that their place code must be an ensemble code, otherwise the activity of individual place cells have an ambiguous interpretation in terms of spatial location (Fenton et al., 2008; Henriksen et al., 2010; Park et al., 2011).

### The hippocampus circuit as an exemplar of neural coordination

Given that the hippocampus expresses an ensemble code, at least for space, then it must utilize neural coordination mechanisms to function and avoid the superposition catastrophe. Electrophysiological recordings from multiple hippocampus neurons and/or sites demonstrate that coordinated activity is accomplished at multiple levels, such as the timing of action potentials, excitation/inhibition balance, and the coordination of oscillatory activity, all rooted in the anatomical connectivity that defines neural circuits (Buzsaki, 2010). Importantly, we observe dynamic grouping of place cell activity during the active place avoidance behavioral paradigm that we use to study cognitive control (Kelemen and Fenton, 2010, 2013) indicating that hippocampal neural coordination is a neural correlate of cognitive control.

For being a relatively simple cortical circuit, the hippocampus is anatomically complex (Figure 1C). The simplistic, traditional version of the circuit is known as the trisynaptic loop, defined as entorhinal cortex ⇒ dentate gyrus ⇒ CA3 ⇒ CA1 ⇒ entorhinal cortex (Andersen et al., 1971). According to the framework first established by Marr (1971), the dentate gyrus is proposed to separate input activity patterns from one another making them more distinctive than they might actually be in the input regions, whereas CA3 is thought to be an autoassociative network that accomplishes the opposite computation, called pattern completion, to attenuate small differences between neural activity patterns. Finally, the CA1 field was conceptualized as a relay to transfer the processed information back to neocortex (Marr, 1971). While each of these information processing steps is important for cognitive function, pattern separation is perhaps the most fundamental for cognitive control and the flexible use of information. It is difficult to understand and act differently in response to two similar experiences if distinctive internal representations are not available to support knowledge and behavior. Consistent with this view, manipulations of the dentate gyrus can improve (Sahay et al., 2011a) or compromise cognitive discrimination and flexibility (Burghardt et al., 2012; Nakashiba et al., 2012).

The contemporary understanding of the hippocampus circuit recognizes that it is substantially more elaborate than the trisynaptic loop suggests (Amaral and Witter, 1989). The entorhinal cortex layer II sends projections to CA3 as well as dentate gyrus, while entorhinal cortex layer III sends direct projections to CA1 (reviewed in Van Strien et al., 2009). Additionally, CA3 and entorhinal cortex send inputs to stratum radiatum and stratum lacunosum of CA1, respectively (Figure 1C). Thus, information coming into CA1 from multiple, anatomically segregated streams must be coordinated to generate coherent and organized CA1 spiking output. Slow gamma (30–60 Hz) oscillations in the CA1 local field potential (LFP) are synchronized with spiking in CA3 and fast gamma (60–100 Hz) oscillations in CA1 are synchronized with medial entorhinal cortex spiking (Colgin et al., 2009). These excitatory pathways are carefully balanced by feedforward and feedback inhibition that is mediated by numerous inhibitory cell types (Freund and Buzsaki, 1996). A number of these interneuron classes are demonstrated to have distinct temporally coordinated relationships to ongoing oscillatory and spiking states of hippocampus activity (Klausberger et al., 2003; Jinno et al., 2007) as well as control of distinct modes of action potential discharge and oscillation-paced spiking (Royer et al., 2012). Thus, the hippocampus circuit is a rich repository of multi-level neural coordination phenomena.

### The discoordination hypothesis

Our studies of the rich neural coordination phenomena in hippocampus during tasks with low (Fenton et al., 2008, 2010) and high cognitive control demand (Kelemen and Fenton, 2010, 2012, 2013) naturally lead to the hypothesis that abnormally coordinated neural activity results in poor cognitive control (Fenton, 2008), which is an idea that has been proposed in a number of contexts (Tononi and Edelman, 2000; Phillips and Silverstein, 2003; Lisman et al., 2010). Neural discoordination may occur due to improper functioning in several aspects: excitation/inhibition balance, timing of oscillatory activity, anatomical connectivity or synaptic network function. It is important to recognize that although the activity and functional specialization of any individual brain region may appear to be relatively normal, the dynamics of the interactions within and among brain regions may be disrupted (Tononi and Edelman, 2000). For example, individual place cell properties within the hippocampus may appear to be unchanged while interactions amongst cells within one hippocampus (intrahippocampal coordination) or between the hippocampi (interhippocampal coordination) could be sufficiently altered to cause cognitive impairment (Lee et al., 2012). We emphasize below that the causes of cognition-impairing discoordination can be very diverse and, consequently, the expression of discoordination can also be diverse. The bulk of prior work on mental illness and animal models has focused on disruptions of “unitary” processes like synaptic plasticity, neurotransmission failure or excess, and abnormally localized function, whereas we wish to advocate for the growing awareness that the interactions amongst relatively intact processes may be abnormal in mental illness (Phillips and Silverstein, 2003). As these interactions become better understood we expect that specific forms of neural discoordination will underlie specific features of cognitive dysfunction. Such biomarkers are likely to be important for differentiating patients with diagnoses of heterogeneous illnesses such as schizophrenia, and most other forms of mental illness, including even those with remarkable heterogeneity of symptoms, despite a specific genetic origin like the fragile × syndrome (Mitchell et al., 2013). Unfortunately it is still early days.

The discoordination hypothesis makes three general predictions that our research program is testing. (1) Subjects with neural discoordination will perform poorly in tasks that require cognitive control. (2) Conversely, subjects with poor cognitive control have neural discoordination. (3) Normalizing neural coordination is sufficient to restore cognitive performance in cognitively impaired individuals, regardless of the etiology of the dysfunction. Put another way, how neural discoordination arises is secondary if we can determine a way to restore the system to the appropriately coordinated state.

## Cognitive control in rodents

As a rat moves across the substrate of an arena that is housed in a lit room, the rat can know its location using three principle sources of information: (1) local (arena) cues, (2) distal (room) cues, and (3) idiothetic cues from its self-motion senses (reviewed in Bures and Fenton, 2000; Fenton and Bures, 2003). This is analogous to our own ability to know locations in a room relative to the furniture, relative to the enclosing walls, and relative to the distance and direction of our own movements. We have repeatedly demonstrated that any one of these three cue sets is sufficient for place avoidance (Bures et al., 1997, 1998; Fenton et al., 1998; Stuchlik et al., 2001; Kubik and Fenton, 2005; Wesierska et al., 2005). Under normal circumstances, all estimates converge on the same location, making it impossible for a researcher to know which cues the animal is using by observing overt behavior. However, slowly rotating the arena dissociates the information from the spatial frames of the arena and room and this allows resolution of the ambiguity. In an early study, rats were trained to avoid a shock zone on a stationary arena. After they had reached their performance asymptote, the shock was turned off and the arena was rotated for the first time in the rat's life. It was only then that we observed that the animals had learned to avoid both the stationary location of shock in the room and the location of shock on the arena, which was now rotating (Fenton et al., 1998).

**Figure 2:**
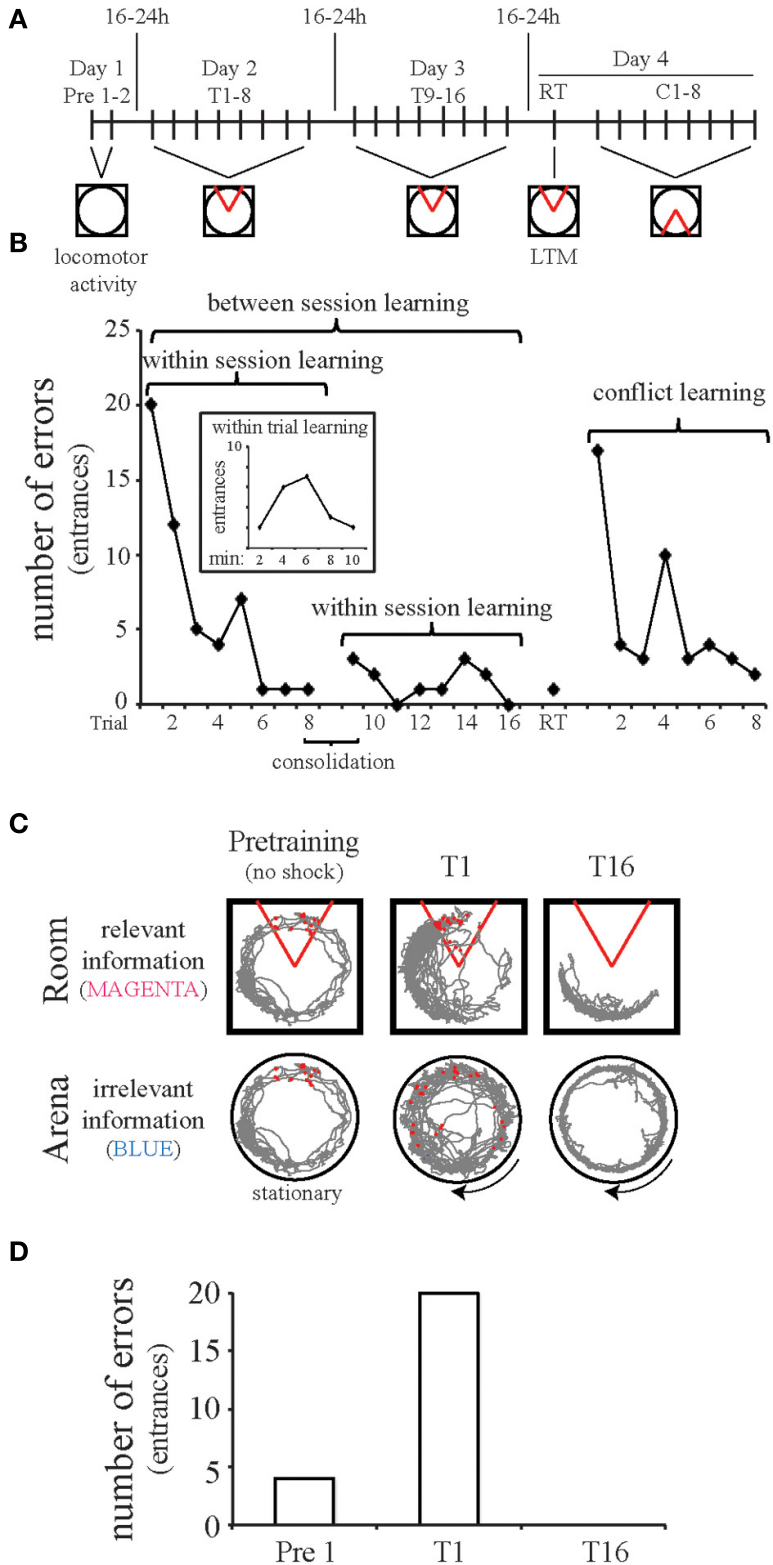
**The active place avoidance paradigm is used to study cognitive control**. The apparatus consists of an 82-cm diameter disk-shaped arena with transparent walls and extra-arena visual cues placed within the room. **(A)** Experimental protocol. Pretraining (Pre 1–2), the animal explores the stationary arena, during which time no shock is delivered. To evaluate active place avoidance, the animals are placed on the rotating arena (1 rpm) and a mild foot-shock (~0.3 mA) is delivered for 500 ms whenever the rat enters the shock zone, which is a 60° sector that is defined by the computer in room coordinates. The shock is repeated every 1500 ms until the subject leaves the shock zone. Avoidance is measured by counting the number of errors, defined as occasions that the animal enters the shock zone. Animals are trained over the course of several days to study learning, memory retention, and cognitive flexibility. Each trial [training 1–16 (T1–16), retention (RT), and conflict learning (C1–8)] is 10 min and the interval between trials is at least 10 min. After training for multiple days, cognitive flexibility is assessed during conflict learning after shifting the shock zone 180° from the original location. **(B)** Behavior of a single rat. The pretraining sessions are open field tests and can be used to assess overall locomotor activity and behavioral habituation in response to novelty. Testing over multiple days allows examination of within-session learning across trials and between-session learning across days. We also assess within-trial learning (inset of graph) over the 10 min trial by assessing the number of errors made during two min periods. For the animal presented in this figure, the learning curve over the first trial shows that the rat received more shocks in minutes 4–6 than in the first 2 min. This pattern is atypical but not uncommon, as avoidance can occur by chance for a few minutes if the animal runs away in response to shock. Avoidance by chance is unlikely for prolonged periods of time, which is why end point measures are taken over a 10 min period or longer. Consolidation can also be examined by comparing the performance at the end of day one to the performance at the beginning of day two. Long-term memory (LTM) is tested in a single retention trial (RT) 1 day after training to the initial shock zone. Reversal learning is examined over eight conflict trials (C1–8) with the shock zone shifted 180°. Rodents quickly learn both the original and reversed shock location. **(C,D)**. The two-frame active place avoidance task resembles the Stroop test, where the room frame represents relevant information (similar to the word color) while the arena frame represents irrelevant information (similar to the word meaning). Place avoidance was measured as the number of entries into the shock zone (errors). Initially (T1), a control animal makes errors. By the sixteenth trail (T16), this animal makes no errors. The data presented in B-D are from the same animal. Red circles indicate shocks. The gray lines are the path of the animal throughout the trial with respect to the Room or Arena frame. The 60° area defined by the red lines is the shock zone, stationary within the room frame.

We devised the active place avoidance behavioral paradigm on a rotating arena to evaluate forms of cognition, including cognitive control in rodents (Bures et al., 1997; Cimadevilla et al., 2000b, 2001a). The standard task variant is configured to present rodents with the cognitively challenging component of the Stroop test for humans. Rodents are exposed to two competing streams of information, one of which must be ignored to successfully avoid a shock zone (Figures 2A–C). The animals are placed on the slowly rotating arena with a stationary shock zone, the location of which is defined within the room coordinates. Because only the room cues provide relevant information for learning and avoiding the location of shock, the animals must not associate shock with locations on the rotating arena. The rotating cues are irrelevant for avoiding shock because these cues do not provide information to predict shock. Normal laboratory rodents quickly learn this standard task variant, reaching asymptotic performance within three 10-min trials (Figures 2B,D). This so called “Room + Arena-” variant of the task requires the dorsal hippocampus (Cimadevilla et al., 2000a) and is one of the most sensitive rodent tasks to hippocampal dysfunction, as it is impaired by even partial, unilateral inactivation of a hippocampus (Cimadevilla et al., 2001b; Wesierska et al., 2005). We have also directly demonstrated task-relevant neural coordination phenomena in single-unit ensemble and LFP studies during performance of place avoidance tasks (Kelemen and Fenton, 2010, 2012, 2013; Lee et al., 2012).

Rotating the experimental arena, as in the standard place avoidance task variant, allowed us to operationally define cognitive control as the ability to selectively interpret and respond to task-relevant stimuli while suppressing competing irrelevant interpretations and stimuli. If all of the sources of information converge on the same place, cognitive control is unnecessary (stable arena in a lit room). When the arena rotates, the spatial frames are dissociated and cognitive control becomes necessary because during learning, each time the rat is shocked, it must select an interpretation. The rat must learn the stationary room location of the shocks and suppress associating the rotating locations of shock with the irrelevant arena locations. Cognitive control is less necessary for the various one-frame task variants such as the (Room&Arena)+ configuration on a stationary arena or the Room+ variant on a rotating arena with shallow water that hides the local cues. Impaired two-frame avoidance and spared one-frame avoidance is pathognomonic of selectively-impaired cognitive control.

## Three schizophrenia-related neurodevelopmental models

Poor cognitive control is one of the most debilitating impairments in schizophrenia and, because it is central to the discoordination hypothesis, it is necessary that animal models valuable for testing the hypothesis have cognitive control deficits. According to the hypothesis, it follows that animal models that do not display impairments in cognitive control will have normal neural coordination. We are testing the discoordination hypothesis using three neurodevelopmental animal models that are considered to be relevant to schizophrenia: (1) a temporary hippocampal inactivation model (Lipska et al., 2002b) in which tetrodotoxin is injected into the neonatal ventral hippocampus (ttxNVHL) on postnatal day 7 (PD7), (2) the permanent neonatal ventral hippocampal lesion (Lipska et al., 1992) caused by injecting the excitotoxin ibotenic acid (iboNVHL) on PD7, and (3) exposure of the developing brain to the toxin methylazoxymethanol acetate (MAM) at gestational day 17 (GD17-MAM) (Lodge and Grace, 2009).

The ttxNVHL and iboNVHL neurodevelopmental models involve direct insults to the developing hippocampus with the intention of altering development of connected brain circuits such as those in the prefrontal cortex, dorsal hippocampus, nucleus accumbens and amygdala (Lipska and Weinberger, 2002). The hippocampus is temporarily inactivated in the ttxNVHL model and permanently damaged in the iboNVHL. TTX inactivation likely lasts about half a day based on studies of the effect of TTX in the adult CNS (Zhuravin and Bures, 1991; Olypher et al., 2006). In both versions of the NVHL model the insult occurs on PD7, a time that is thought to correspond to the third trimester of pregnancy in the course of human brain development (Rakhade and Jensen, 2009; Sengupta, 2011). It is important that the insult occur at an early age to sufficiently impact the development of brain circuits. Performing the lesion in an adult hippocampus does not result in the same schizophrenia-related abnormalities (Lipska et al., 2002a; Tseng et al., 2009) implying there is a developmental component to the consequences of the NVHL procedure. The impact of MAM on the developing brain is nonspecific but a comprehensive review of identified changes is available (Lodge and Grace, 2009). Among brain regions affected by MAM are the prefrontal cortex (Esmaeili and Grace, 2013), nucleus accumbens (Perez and Lodge, 2012), ventral hippocampus (Lodge and Grace, 2007), and the ventral subiculum-nucleus accumbens-prefrontal cortex circuit (Belujon et al., 2014). Histological assessment of hippocampi in GD17-MAM animals shows morphological deficiencies (Matricon et al., 2010) that we have confirmed in unpublished studies. These deficiencies include a thinned pyramidal cell layer with disruptions in the CA1 and CA3 subfields. It may be important that the timing of MAM administration coincides with peak neurogenesis in the CA1 and CA3 subfields during embryogenesis (Schlessinger et al., 1978; Bayer, 1980).

## Active place avoidance in the three neurodevelopmental models

**Figure 3:**
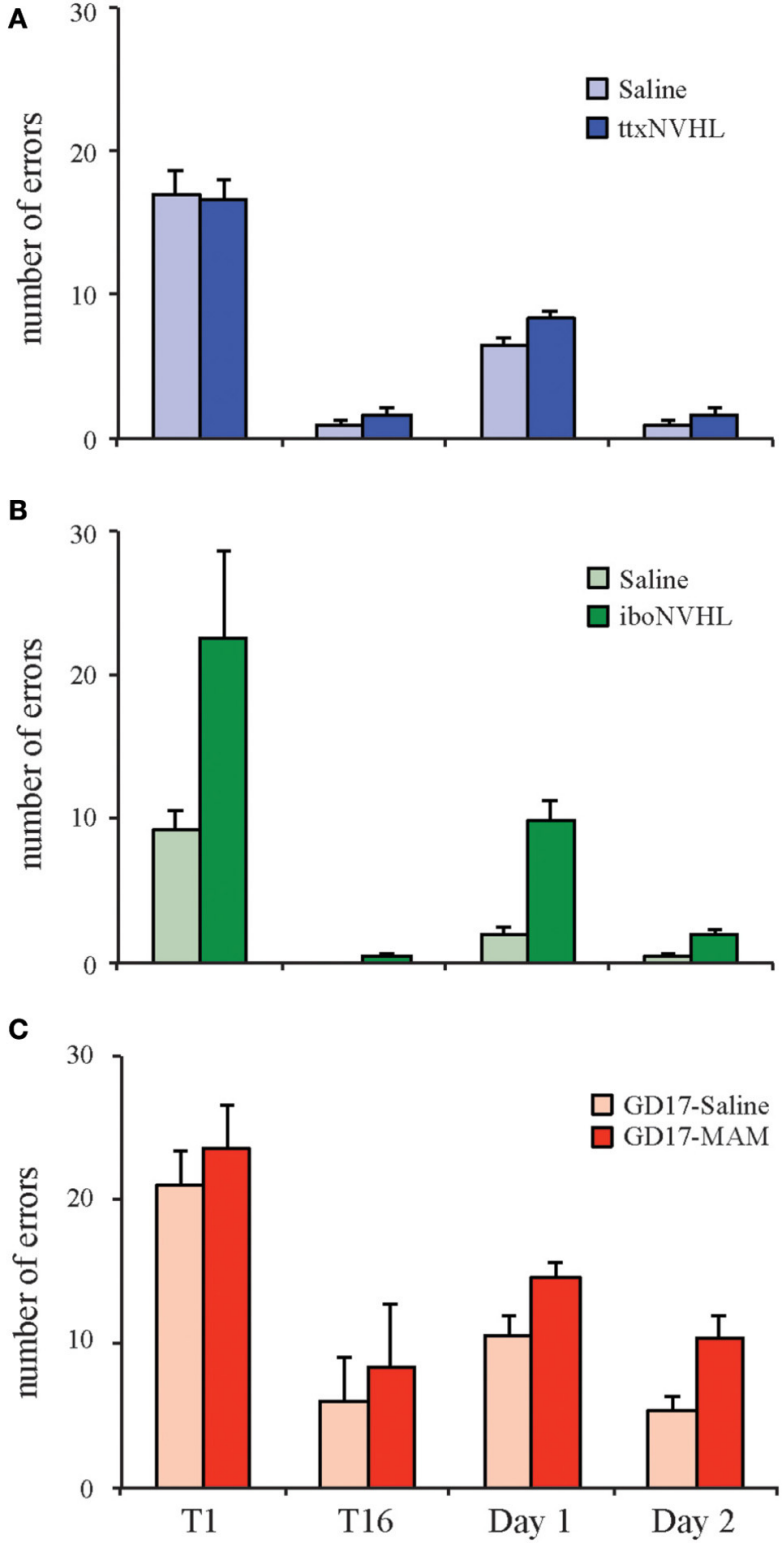
**Neurodevelopmental insults differently affect cognitive ability**. Three animal models of schizophrenia are generated in the Long Evans strain of rats and are being used to test the neural discoordination hypothesis. Cognitive control was tested using the two-frame active place avoidance task. Generation of the ttxNVHL and iboNVHL is as follows: on postnatal day 7, male pups were anesthetized by hypothermia and bilateral injections of TTX (30ng/μL; 0.3 μL/hemisphere), ibotenic acid (10 μg/μL; 0.3 μL/hemisphere), or an equal volume of saline was injected into each ventral hippocampus. To generate MAM animals, timed pregnant females were given intraperitoneal injection of MAM (26 mg/kg) or an equal volume of saline at gestational day 17 (GD17). **(A)** ttxNVHL animals do not have cognitive deficits in the two-frame active place avoidance task and are predicted to have normal neural coordination. **(B)** Adult iboNVHL animals have deficits in the two-frame active place avoidance task and also have altered interhippocampal neural coordination (Lee et al., 2012). **(C)** GD17-MAM animals have difficulty performing the two-frame active place avoidance task and are predicted to have poor neural coordination. Data are presented as average ± s.e.m.

We are interested in finding diverse sets of conditions that converge on the canonical target deficit in cognition because the discoordination hypothesis predicts that neural coordination abnormalities accompany cognitive control deficits. We used male Long-Evans rats for the studies discussed here. Adult ttxNVHL animals do not display cognitive deficits in the active place avoidance task (Figure 3A). It is unlikely that the unimpaired performance in the ttxNVHL group is due to a lack of overt lesion since ventral hippocampus is not necessary for spatial learning (Kjelstrup et al., 2002), including active place avoidance. The reason for the lack of ttxNVHL effect on cognitive control is uncertain, though we suspect the insult did not sufficiently alter neural circuit development. While measurements have yet to be done, the discoordination hypothesis predicts there will be normal neural coordination in these animals. In contrast, adult iboNVHL animals display poor cognitive control (Figure 3B) and have abnormal neural coordination while performing the active place avoidance task, consistent with the discoordination hypothesis (Lee et al., 2012, 2014). Similar to iboNVHL animals, adult GD17-MAM animals have deficits in the active place avoidance task (Figure 3C), and according to the discoordination hypothesis, neural discoordination is expected in the GD17-MAM model, especially while the animals are performing cognitively challenging tasks. Accordingly, we are focusing on the iboNVHL and GD17-MAM model for further investigation.

### Cognitive flexibility in the active place avoidance task

**Figure 4:**
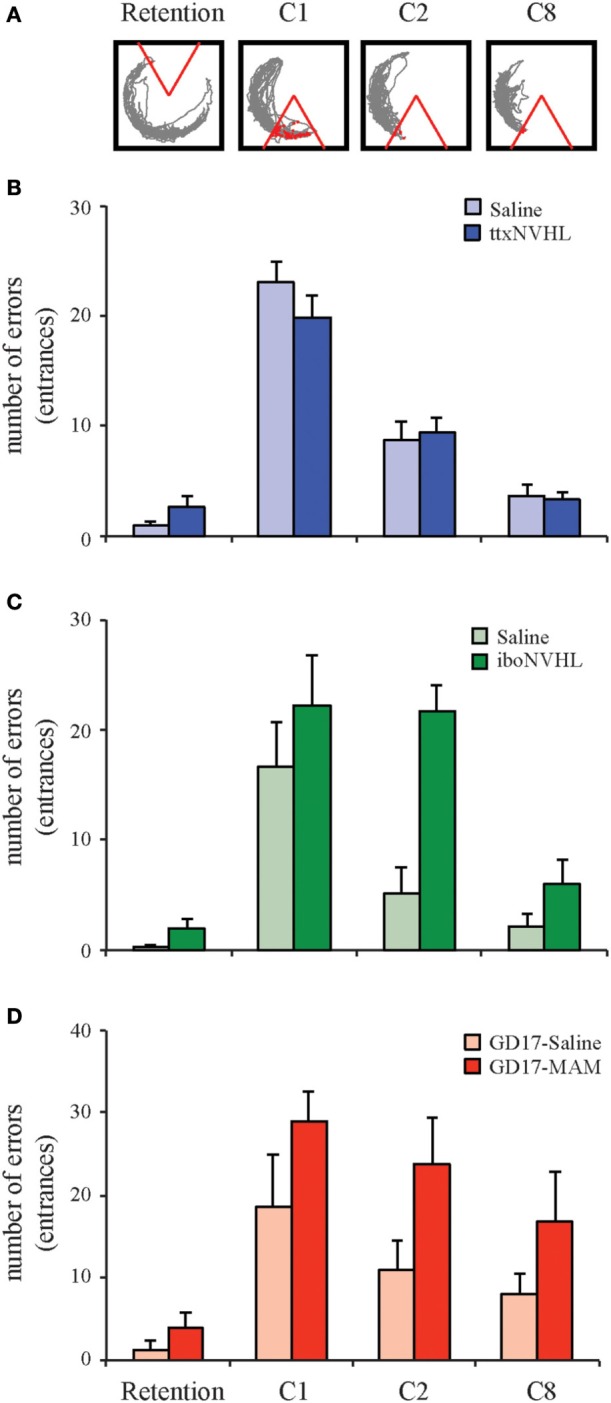
**Cognitive flexibility is differently altered by neurodevelopmental insults**. Active place avoidance can be used to test cognitive flexibility. **(A)** One day after training to the initial shock zone, the animals are tested in a retention trial and eight trials in which the shock zone is shifted 180° from the initial location. The ability to adapt to the conflict between the shifted shock zone location and the memory of the initial shock zone location was measured as the number of entries into the shifted shock zone location (errors). **(B)** Cognitive flexibility is normal in ttxNVHL animals. **(C,D)** As indicated by the retention trial, both the iboNVHL and GD17-MAM rats are capable of performing the active place avoidance task after 2 or 4 days of training, respectively. However, cognitive flexibility during the 180° conflict trials is disrupted in iboNVHL and GD17-MAM animals. Data are presented as average ± s.e.m.

One common feature of the iboNVHL and GD17-MAM neurodevelopmental models is that animals appear to have impaired active place avoidance learning, but with continued training they can eventually perform the task (Figures 3B,C, 4C,D). After the animals have established asymptotic performance, we can further test cognitive control, specifically cognitive flexibility, by shifting the shock zone 180° from the original location. In these conflict trials, control and ttxNVHL animals quickly acquire the ability to avoid the new shock zone, while iboNVHL and GD17-MAM animals make many more errors (Figure 4). Thus, the initial training was not sufficient to overcome the impairment in these two models and perhaps, conflict learning itself was impaired. Previous work showed that mice learned the active place avoidance task as well as controls after ablation of adult neurogenesis in the dentate gyrus by either X-irradiation or a genetic manipulation (Burghardt et al., 2012). Interestingly, the same mice were impaired on the 180° conflict trials from the initial location. They were impaired in that they continued to avoid the initial shock zone location. This cognitive inflexibility caused them to enter the current shock zone often because this location had been their preferred location for avoiding shock in the initial zone. Further, these mice had no difficulty to extinguish the initially learned avoidance when the shock was turned off. This intact extinction learning indicates the deficit was specific to being able to discriminate between the representations of the initial and new shock zone locations and express flexible behavior in response to the changed shock-location contingency.

An important feature of the dentate gyrus is the existence of neurogenesis into adulthood. These findings with place avoidance contribute to mounting evidence for a specific role of adult neurogenesis and the dentate gyrus in cognition. One hypothesis is that recently matured adult born granule cells modulate the excitability of the dentate gyrus network so that it can more easily express differential patterns of activity in response to similar conditions that differ in subtle but important ways, such as the changed location of transient shock (Sahay et al., 2011b; Ikrar et al., 2013). An alternative proposal is that newly matured granule cells preferentially participate in the encoding of memories and thus as time passes, different neurons will represent different information and events (Aimone et al., 2010; Deng et al., 2010). Both hypotheses recognize a role for adult born neurons in pattern separation-dependent learning. The pattern separation computation is thought to underlie the generation of distinctive neural representations of similar items, which, by averting overgeneralization and catastrophic interference at the level of neural representations, can promote effective cognitive discriminations.

## The neurodevelopmental opportunity

Prolonged development of the brain does not permit a simple definition of immature or mature. The formation of neural circuits, in the most basic form, is established by neurogenesis *in utero*. However, although these foundational neural circuits are present at birth, substantial refinement occurs until adulthood. The complex development of brain circuits and functions has been discussed in comprehensive reviews, such as these examples that consider prolonged changes in both brain organization and behavioral expression (Bachevalier and Beauregard, 1993; Rice and Barone, 2000). Here we will focus on a subset of details that concern the hippocampus in the effort to advance our argument that a developmental perspective is valuable for conceptualizing and investigating mental illness. In the hippocampal formation, prolonged development into the postnatal period is evident in several aspects and the timing of some major developmental processes have been summarized in Table 1.

**Table 1:**
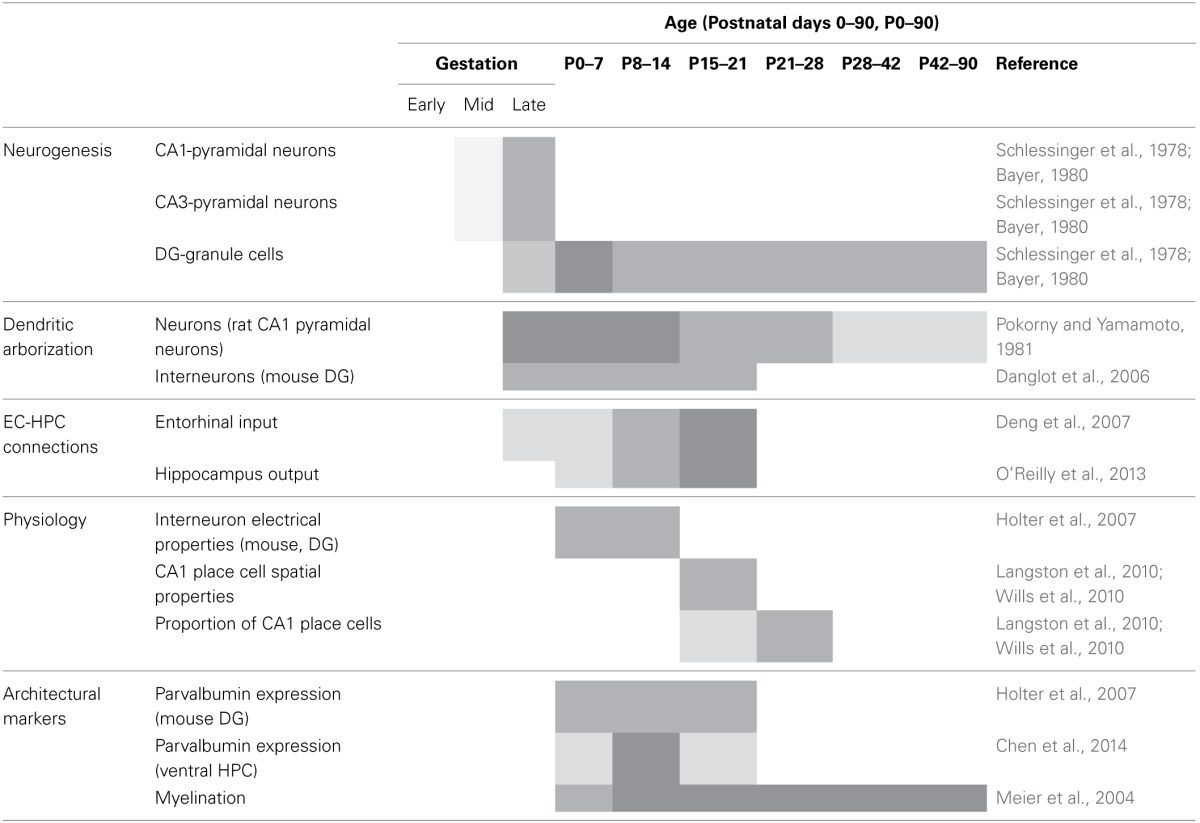
**Prolonged developmental processes of the hippocampal region in rodents**.

*Shaded areas correspond to changes during development. The darker the color, the larger the change is within that row, but do not correlate to magnitude of changes with respect to other rows. In categories where changes seemed even across development, the middle shade of gray was selected. Data is from rats unless indicated otherwise. CA1, cornu ammonis; reDG, dentate gyrus*.

These prolonged developmental processes create a window of vulnerability for negative or positive impact on adult cognitive ability because the functional refinement of neural circuits establishes the foundation upon which future coordinated and temporally sculpted neural activity is based (Buzsaki, 2010). This window of vulnerability is the basis for the two-hit hypothesis of precipitating schizophrenia and other mental illness when environmental factors have a negative impact on cognitive circuits during adolescence. As such, schizophrenia is hypothesized to be a neurodevelopmental disorder (Weinberger, 1987, 1996; Insel, 2010) and certainly the increased odds of the disorder given a neonatal insult suggests some kind of developmental process is involved (Weinberger, 1987). The ttxNVHL and gestational exposure to MAM produce absent or strong forms of cognitive control impairment, respectively, indicating that development must be taken seriously in the realm of cognition. Importantly, the developmental insult doesn't negatively impact everything, as evidenced by the fact that the animals with neurodevelopmental insults can still see, locomote, and learn in many domains, like controls. Adult iboNVHL rats have normal forms of hippocampus-dependent memory (Beninger et al., 2009). Normal learning was also demonstrated in the place avoidance paradigm because adult iboNVHL were indistinguishable from control rats in the task when the arena was covered with shallow water to attenuate the irrelevant arena-frame spatial cues (Lee et al., 2012).

**Figure 5:**
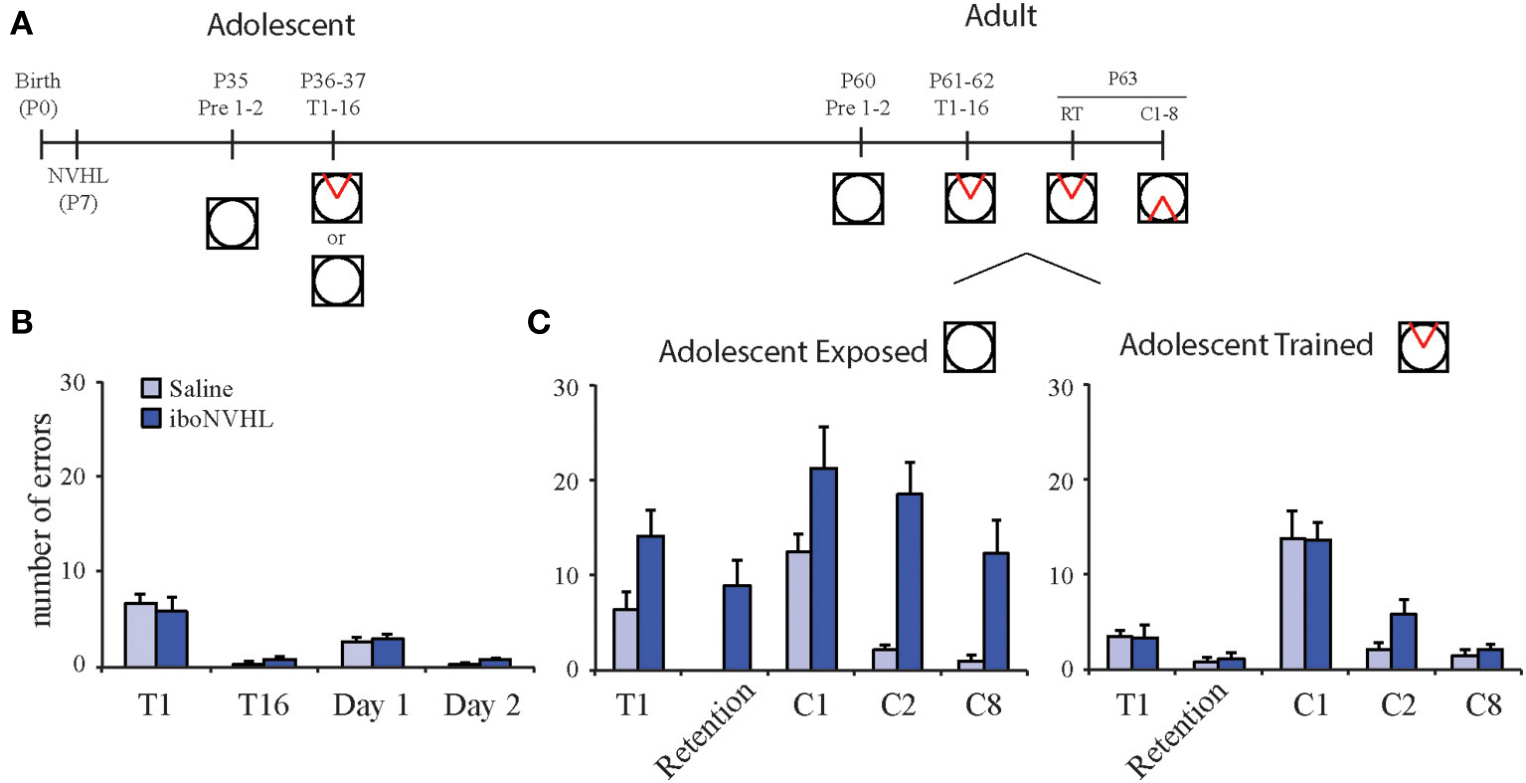
**Adolescent experience alters adult behavior in iboNVHL animals. (A)** At postnatal day 35 (P35), iboNVHL animals were habituated to the room on the stationary arena for two 10 min sessions with a 10 min inter trial interval. Adolescence training consisted of eight trials per day for 2 days (P36 and P37). To control for noncognitive experience, a group of adolescent animals was received the same experience with the shock off. **(B)** During adolescence, iboNVHL animals are able to perform the task as well as saline controls. **(C)** Adolescence training had a positive impact on adult behavior in the active place avoidance task. iboNVHL animals that received cognitive training during adolescence performed as well as saline control animals when tested as adults. The animals that received noncognitive experience during adolescence remained impaired. Data are presented as average ± s.e.m.

We are evaluating the idea that it is possible to take advantage of the prolonged development in order to correct the cognitive impairments, or at least improve outcomes, by modifying neural circuit refinement. We examined the impact of cognitive training during adolescence on adult active place avoidance (Figure 5). When tested during adolescence, the iboNVHL animals were capable of learning the active place avoidance task as well as control animals (Figure 5B). Remarkably, iboNVHL animals that were trained during adolescence performed as well as control animals during adulthood in the initial learning and conflict learning variants of the active place avoidance task (Figure 5C). These benefits seemed to generalize broadly because the adolescence training also normalized reversal learning in a T-maze (Lee et al., 2012). In contrast, iboNVHL animals still showed cognitive impairments as adults if they were merely exposed to the rotating arena without any explicit cognitive demand during adolescence (Figure 5C). These cognitive behavioral benefits were paralleled by normalization of the synchrony of LFP oscillations between the two hippocampi, which directly measures neural coordination. Thus, cognitive experience in adolescence is demonstrated to improve both cognitive performance and neural coordination. These findings establish a rationale for investigating whether appropriately timed preemptive cognitive training can be therapeutic. We are naturally interested in examining the generality of these findings and are investigating whether preemptive cognitive training in adolescence can also improve cognition and neural coordination in the GD17-MAM model.

## Possible targets to therapeutically improve neural coordination and cognitive outcomes

**Figure 6:**
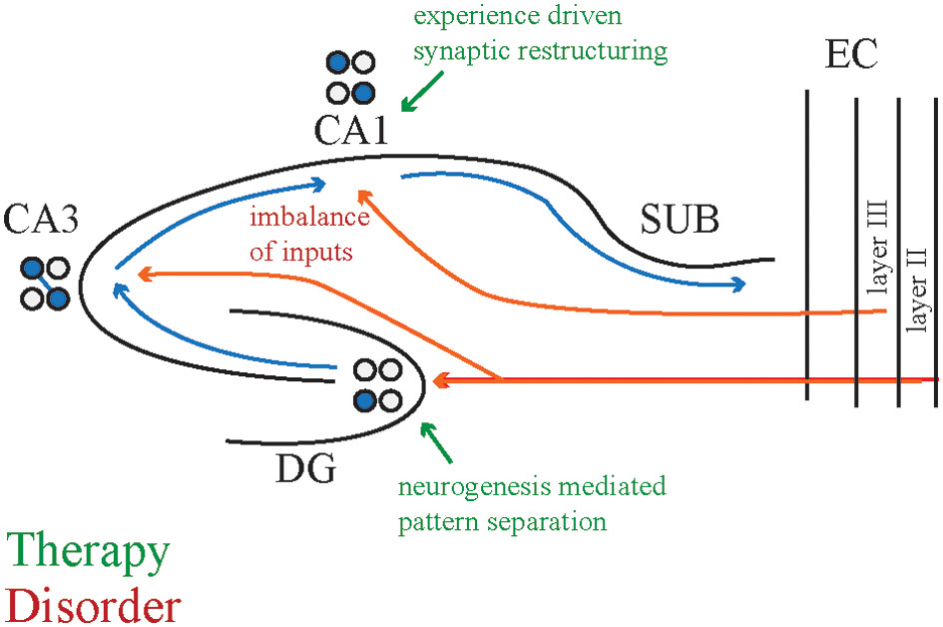
**Potential circuit modifications to attenuate cognitive deficits**. One hypothesized cause of cognitive deficits is an imbalance between the entorhinal cortex and CA3 inputs to CA1 that may carry perceptual and memory related information, respectively. Cognitive training may induce synaptic restructuring, for example by increasing the CA3 input and decreasing the entorhinal input to normalize CA1 responses. A second hypothesized cause of cognitive deficits is the abnormal pattern separation computation that might underlie cognitive flexibility deficits. Cognitive training may increase the survival:death ratio of newly born neurons in the dentate gyrus to promote better pattern separation. The adolescent brain may be more receptive to these changes than the adult brain because juvenile brains are actively undergoing similar modifications. CA1, cornu ammonis 1 subregion; CA3, cornu ammonis 3 subregion; DG, dentate gyrus; SUB, subiculum; EC, entorhinal cortex.

We are investigating potential mechanisms by which preemptive early cognitive training can correct neural coordination and cognition in models of mental dysfunction. As our ongoing investigations focus on the hippocampus, we discuss the hippocampal circuit (Figure 6), but suggest that this perspective on neural circuit computations may be advantageous for considerations of many circuits involved in cognition.

CA1 receives converging, temporally coordinated inputs from CA3 and entorhinal cortex (Colgin et al., 2009), and we have observed discoordination in these signals in genetic and pharmacological animal models with active place avoidance deficits (unpublished observations). If synaptic function is not appropriately weighted between these two regions, we hypothesize that cognitive training can adjust relative synaptic functional strengths within the neural circuit in a use-dependent manner. According to this synaptic plasticity hypothesis, we speculate that the appropriate cognitive training can refine and tune the circuit so that it operates in an improved functional state. This synaptic restructuring promotes neural coordination in CA1 in support of cognitive control and flexibility. In addition to synaptic plasticity mechanisms, cognitive training may alter neural circuit and synaptic network function by changing the expression of dendritic spine structure (Yang et al., 2009) and/or structural proteins (Gray et al., 2006; Clem et al., 2008; Wen et al., 2013). In fact, we have observed that place avoidance training in adolescence decreased parvalbumin expression in mPFC interneurons (Lee et al., 2012).

As another potential therapeutic target, we have also considered neurogenesis in the dentate gyrus, which continues past postnatal day 18 (Bayer, 1980) and can be modified throughout adulthood by environmental enrichment and experience (Kempermann et al., 1997; Van Praag et al., 2005; Mirescu and Gould, 2006; Zainuddin and Thuret, 2012; Gregoire et al., 2014). As discussed above, adult dentate gyrus neurogenesis has a role in cognitive flexibility, measured by the place avoidance paradigm. At 1 month of age, dentate gyrus neurogenesis is higher and apoptosis is lower than at 3 months of age in the rat (Silva et al., 2006). Experiences that alter adult dentate gyrus neurogenesis may especially do so during adolescence. Thus, the adolescent cognitive training that normalized cognition and related neural coordination may have adjusted the balance between neurogenesis and apoptosis, promoting better pattern separation and completion computations within the hippocampal circuit.

Schizophrenia, and other forms of mental illness present some of the most difficult medical problems to investigate because of the diverse and unknown etiology of the disorders. Our effort focuses on cognitive control, a key symptom, and evaluates how multiple animal models impact that ability. The goals are to converge on a common pathophysiology that is important for the cognitive deficit, independent of the experimental manipulation, and to identify ways in which to alter and attenuate this pathophysiology. We are a long way from obtaining those goals but an optimistic possibility that this effort has suggested is that neurodevelopmental vulnerability can be exploited and harnessed by cognitive experience to improve cognitive outcomes later in life.

### Conflict of interest statement

The authors declare that the research was conducted in the absence of any commercial or financial relationships that could be construed as a potential conflict of interest.
